# Plin5 Bidirectionally Regulates Lipid Metabolism in Oxidative Tissues

**DOI:** 10.1155/2022/4594956

**Published:** 2022-03-31

**Authors:** Xinqing Zhang, Wu Xu, Rui Xu, Zhen Wang, Xinyan Zhang, Peng Wang, Ke Peng, Meiling Li, Jing Li, Yanfei Tan, Xiong Wang, Haifeng Pei

**Affiliations:** ^1^College of Medicine, Southwest Jiaotong University, Chengdu 610031, China; ^2^School of Medicine and Life Sciences/Reproductive and Women-Children Hospital, Chengdu University of Traditional Chinese Medicine, Chengdu 611137, China; ^3^Department of Cardiology, The General Hospital of Western Theater Command, Chengdu 610083, China; ^4^Department of Medical Information Service, The General Hospital of Western Theater Command, Chengdu 610083, China; ^5^National Engineering Rsearch Center for Biomaterials, Sichuan University, Chengdu 610065, China

## Abstract

Cytoplasmic lipid droplets (LDs) can store neutral lipids as an energy source when needed and also regulate the key metabolic processes of intracellular lipid accumulation, which is associated with several metabolic diseases. The perilipins (Plins) are a family of proteins that associate with the surface of LDs. As a member of Plins superfamily, perilipin 5 (Plin5) coats LDs in cardiomyocytes, which is significantly related to reactive oxygen species (ROS) production originated from mitochondria in the heart, consequently determining the progression of diabetic cardiomyopathy. Plin5 may play a bidirectional function in lipid metabolism which is in a state of dynamic balance. In the basic state, Plin5 inhibited the binding of comparative gene identification-58 (CGI-58) to adipose triglyceride lipase (ATGL) by binding CGI-58, thus inhibiting lipolysis. However, when the body is under stress (such as cold, fasting, exercise, and other stimuli), protein kinase A (PKA) phosphorylates and activates Plin5, which then causes Plin5 to release the binding site of CGI-58 and ATGL, prompting CGI-58 to bind to ATGL and activate ATGL activity, thus accelerating the lipolysis process, revealing the indispensable role of Plin5 in lipid turnover. Here, the purpose of this review is to summarize the present understanding of the bidirectional regulation role of Plin5 in oxidative tissues and to reveal its potential role in diabetic cardiomyopathy protection.

## 1. Introduction

Obesity, diabetes mellitus, dyslipidemia, and hypertension often cluster together as the most significant risk factors for cardiovascular diseases [1]. Excess accumulation of intracellular lipid is believed to cause several metabolic diseases like obesity cardiomyopathy, inducing irreversible damage to cardiovascular systems [2, 3]. The rising trend in the lipid-associated metabolic diseases has drawn people's attention to the pathobiological functions of cytosolic LDs which are subcellular structures to store neutral lipid [4, 5]. Medical treatment to prevent against excess accumulation of intracellular lipid alleviated the adverse development of cardiovascular diseases [6], which appeared to be controlled by LDs-associated proteins. Cytosolic LDs are comprised of a core of neutral lipids, triacylglycerols (TAG), and/or cholesteryl ester (CE) and surrounded by a phospholipid monolayer [7]. The contrasting chemical natures of hydrophilic lipid metabolic enzymes and their hydrophobic substrates have directed people's attention to LDs surface which was viewed as regulatory interface between the aqueous cytosol and the hydrophobic lipid core [8]. Thus, LDs may have essential protective roles in the sequestration of cytotoxic fatty acids (FAs) in nonadipose tissues. Specifically, perilipin proteins (Plins) are the definitive abundant proteomic markers of LDs surfaces in both adipose and non-adipose cells and function as primary mediators for neutral lipid storage/hydrolysis [9]. LDs are coated by several proteins, including Plins and other structural proteins, membrane-trafficking proteins, lipases, and lipogenic enzymes [10]. Especially, Plins are known as representation of LDs and necessarily associated with LDs formation [11]. The mammalian genome has encoded five Plins genes, with unique tissue-dependent patterns of transcription and splice variation [12]. Perilipin 1 (Plin1) is abundant in white adipose tissue (WAT) and brown adipose tissue (BAT). Perilipin 2 (Plin2) and perilipin 3 (Plin3) are widely distributed, with Plin2 highly expressing in hepatocytes. Perilipin 4 (Plin4) is observed in adipocytes, cardiomyocytes, and myocytes. However, Plin5 is highly expressed in oxidative tissues and generally restricted to tissues/cells that utilize lipids for energy through mitochondrial *β*-oxidation. [13, 14]. Overall, Plins take important roles in the formation and degradation of LDs.

**Figure 1:**
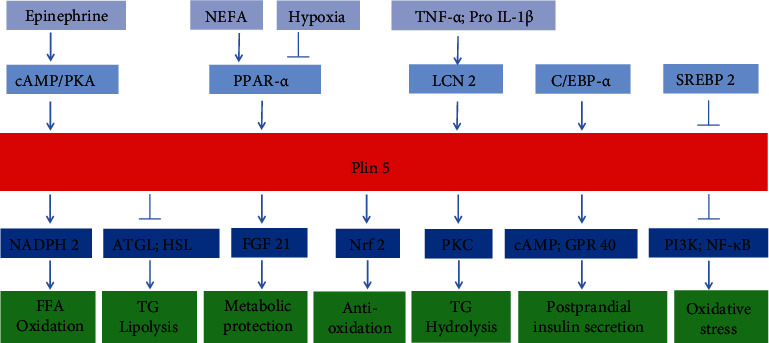
The upstream and downstream regulating factors for plin5.

As a member of the perilipin superfamily, Plin5 is central to lipid homeostasis in these tissues by promoting association of LDs with mitochondria [15]. Besides the expression on surface of LDs, Plin5 also appears in nucleus, cytoplasm, and mitochondria. Plin5 is closely related to the generation of ROS originated from mitochondria, consequently determining the progression of oxidative stress [16]. Recently, the data from *in vivo* and *in vitro* indicate an important role of Plin5 in the regulation of cardiac lipid storage and function. Previous studies have suggested that lipolysis was mainly due to PKA -mediated phosphorylation of hormone-sensitive lipase (HSL), but recent studies have shown that it is mainly regulated by the translocation of lipase from the cytosol to the surface of LDs. And Plin5 phosphorylation is necessary for HSL translocation and has become a hot topic in recent years due to the important role of Plins in lipolysis. Under basal conditions in cardiac tissues, Plin5 coats on the surface of LDs as a physical barrier to inhibit enzymatic activity, inhibiting the binding of CGI-58 to ATGL by binding CGI-58, thus inhibiting lipolysis, as well as a guarder to prevent excessive *β*-oxidation of free fatty acids. When phosphorylated in lipolysis following activation of PKA under *β*-adrenoceptor-stimulated condition or when energy demand is increased, Plin5 serves as a platform to facilitate lipolysis on LDs surface by promoting the interaction of ATGL with CGI-58, thus accelerating the lipolysis process [17]. Moreover, besides the promotion of *β*-oxidation, phosphorylated Plin5 can migrate to nucleus to promote peroxisome proliferator-activated receptor gamma coactivator 1-*α* (PGC-1*α*) function by disinhibiting sirtuin 1 (SIRT1) deacetylase activity, enhancing transcription of mitochondrial function, and reinforcing fatty acid metabolism. Therefore, Plin5 may serve as a bidirectional regulator for lipid turn over by phosphorylation modification and content variation in cardiomyocytes and act as a potential molecular target for the treatment of dyslipidemia in diabetic cardiomyopathy. However, in skeletal muscle, the phosphorylation level of Plin5 remains unchanged when facing with either contractile or adrenergic stimulation, revealing that Plin5 may share different functions in different tissues (Figure 1). The focus of this review was to summarize the reported roles of Plin5 in oxidative tissues.

## 2. Special Function of Plin5 in Oxidative Tissues

### 2.1. Cardiac Tissues

In contrast to other perilipins, Plin5 has unique ability to store lipid droplet in O_2_ abundant organelles. Studies have shown that free fatty acid (FFA) can be used as ligand to activate peroxisome proliferator-activated receptors (PPARs) and induce the expression of Plin5. For example, exogenous FFA can stimulate the expression of Plin5 in cultured rat cardiomyocytes [18]. Conversely, Plin5 can also affect the metabolic process of FFA. It is found that the FFA uptake decreased and glucose uptake increased after the knockout of Plin5 in myocardium, which thus maintained the normal energy requirement of the heart [19], and that the expression of FFA uptake-related enzymes such as lipoprotein lipase (LPL) and CD36 was decreased in myocardial overexpressing Plin5 [20]. This result suggested that the increase in TAG during Plin5 overexpression is not likely due to increased FFA uptake. Besides, Plin5 could regulate the *β*-oxidation of FFA by acting on the spatial action of mitochondria and affecting FFA-related enzymes. Research found that the expression of mitochondrial oxidation-related genes and Plin5 were negatively correlated in myocardium [20, 21]. And Plin5 ablation could enhance the oxidation of FFA in the myocardium [22]. Further research found that the mRNA expression of mitochondrial cytochrome C and cytochrome C oxidase subunit IV (COX IV) in myocardium of Plin5^−/−^ mice increased significantly compared with WT mice, indicating that mitochondrial function was enhanced [19]. On the other hand, overexpression of Plin5 decreased the expression of PPARs target gene in myocardium [20], and the gene expressions of mitochondrial energy metabolism, oxidative phosphorylation, and utilization of FFA were all downregulated, which had been reported as PPAR target genes [23]. In another report, myocardial overexpression of Plin5 reduced the expression and activity of carnitine palmitoyltransferase 1 (CPT-1), a key enzyme that regulates FFA entry into mitochondria, showing that mitochondrial uptake of FFA was decreased [21]. In summary, recent studies have shown that Plin5 deletion could increase myocardial FFA absorption and enhance mitochondrial oxidation of FFA; in contrast, overexpression of Plin5 could limit mitochondrial oxidation of FFA.

In cardiomyocytes, mitochondria comprise more than 30% of cell volume and generate about 90% of the ATP [24]. Accordingly, mitochondria are also the major source of ROS in the cardiovascular diseases [24, 25]. ROS is known to be implicated in the induction of cardiac hypertrophy in various pathologic states. Overexpression of Plin5 in cardiomyocytes can increase intracellular ROS levels and content of malonaldehyde (MDA) [21]. Meanwhile, overexpression of Plin5 triggers an upregulation of the NF-E2-related factor 2 (Nrf2) antioxidative pathway with specific increases in gene expression involved in glutathione metabolism [21]. Under the normal condition, Plin5 knockout had no significant effect on the contents of ROS, but in the ischemia-reperfusion injury, Plin5 knockout led to the increase of ROS [26]. Cultured cardiomyocytes from Plin5^−/−^ mice had more actively oxidized FFAs and ROS production than those of WT mice, which was, however, reduced by the administration of an antioxidant N-acetylcysteine [22]. Other studies have found that Plin5 deficiency in hypoxic cardiomyocytes exposed to LDL dramatically increases the levels of unpacked FFA and ROS [27]. It was hypothesized that cardiac ROS production in Plin5^−/−^ mice might result from a surplus flux of FA into the mitochondria. In a study involving type I diabetes, the results were just the opposite: compared with WT, Plin5^−/−^ mice did not exhibit excessive ROS generation or cardiac dysfunction but had an improvement in heart function although LDs decomposition increased [27]. The authors have found that diabetic Plin5^−/−^ mice are resistant to type 1 diabetes-induced heart malfunction due to the suppression of the diacylglycerol/ceramide-PKC pathway and of excessive ROS generation by nicotinamide adenine dinucleotide phosphate (NADPH) oxidase [28]. These seemingly contradictory findings may be due to the specificity of the research models.

We find that excessive LDs are stored in nonfat tissues, such as the liver, pancreas, and coronary arteries, which are often associated with fatty liver, T2DM, and coronary atherosclerotic heart disease [29, 30]. Studies have shown that the increase of intracellular LDs can activate oxidative stress [31]. However, most of the data show that TGs mainly have storage function, and the main toxicity is caused by FFA and its metabolites, such as ceramide and diacylglycerol [32]. Therefore, LDs may improve cytotoxicity more than as a causative agent. In heart, LDs can isolate FFA in the form of TAG to prevent lipid damage induced by FFA and its derivatives [33]. Research shows that Plin5 plays an important role in the regulation of lipid metabolism by LDs in cardiomyocytes. In addition, it has been reported that Plin5 knockout has almost no effect on cardiac function under the basal state [19, 26, 28]. However, other studies have shown that Plin5^−/−^ may aggravate age-related cardiac dysfunction, and this heart defect can be prevented by antioxidant therapy [22]. Meanwhile, in Plin5 overexpression mice, myocardial steatosis, increased heart weight, left ventricular hypertrophy, and mild cardiac function were observed [20, 21]. In the mouse model of myocardial ischemia reperfusion injury, Plin5 deficiency aggravates the heart dysfunction [34], and similar results also were found in another myocardial ischemia model [19]. In a study of Plin5 gene in patients with clinical myocardial infarction, Plin5 gene mutation is related to cardiac dysfunction after myocardial infarction [19]. In a word, these studies suggest that Plin5 plays an important role in maintaining normal cardiac function under normal physiological conditions or pathophysiological conditions.

In summary, the studies on various gene models of Plin5 indicate that Plin5 is required for normal cardiac metabolism and function, but too much Plin5 may lead to cardiomyopathy [17]. And both ablation and overexpression of Plin5 have given rise to harmful results; its concrete function may depend on the progression of cardiac diseases and different damage factors. Therefore, we need to further validate the role of Plin5 in the heart on human diseases.

### 2.2. Hepatic Tissues

Hepatocytes are parenchymal cells of the liver responsible for mobilizing lipids for energy and storing excess lipids in the form of LDs. Excessive accumulation of LDs is the cause of hepatic steatosis; meanwhile, accumulative evidence has suggested that LDs proteins were involved in the pathophysiology of liver diseases characterized by excessive lipid accumulation in hepatocytes, such as alcoholic liver disease, nonalcoholic fatty liver disease (NAFLD), and hepatitis C virus infection [11]. Plin5 is closely related to lipid metabolism, since the abnormal lipid metabolism plays an important role in the liver cell lesions. Previous work has indicated that there was an increase of Plin5 expression in mouse models of NAFLD [35]. Another study shows that cells overexpressing Plin5 release lower amounts of FFAs in basal conditions [36]. All those indicate that Plin5 may contribute to the formation of liver steatosis possibly through inhibition of the release of FFAs from LDs [37]. In Plin5 deficient mice, TG content was found to decrease both in primary mouse hepatocytes and in the liver, implying the involvement of Plin5 in TG accumulation [38]. The above data suggest that Plin5 can prevent the FFA and its metabolites over accumulation, thus preventing liver toxicity damage, but in other Plin5^−/−^ mice did not show up liver injury [39]. Therefore, Plin5 is likely to be critically involved in the development of pathologies associated with fatty liver. However, whether it is in agreement with the situation in human should be further explored.

### 2.3. Muscle Tissues

Skeletal muscle stores significant amounts of TG within intracellular LDs, which are near the mitochondria and endoplasmic reticulum [40]. Plin5 is highly expressed in skeletal muscle [41] and plays a critical role in coordinating skeletal muscle TG metabolism and LDs accumulation [13], which impacts sphingolipid metabolism, and is requisite for the maintenance of skeletal muscle insulin action [42, 43]. At present, there are many studies to explore the role of Plin5 in human muscle tissue. Although Plin5 expression is increased in the muscle and liver of mice fed on high-fat diet (HFD) [35, 44, 45], there is no change of Plin5 expression in skeletal muscle of humans fed on HFD for 12 weeks [46], and most studies report normal Plin5 protein content in obese humans [47, 48]. Consistent with these observations, the expression or content of Plin5 in skeletal muscle does not differ between T2DM patients and normal glycemic individuals [48]. In rat muscle, Plin5 content is correlated with mitochondrial respiration rates on a lipid-derived substrate [49]. The overexpression of Plin5 in skeletal muscle promoted expression of a cluster of genes under control of PPAR-*α* and PGC-1*α* involved in FFAs catabolism and mitochondrial oxidation [50], suggesting the role of Plin5 in mediating the skeletal muscle oxidative gene expression, either directly or indirectly.

### 2.4. Adipose Tissues

There are two kinds of adipose tissue in the human body, including WAT and BAT. WAT acts as an energy depot by storing lipids that are released into circulation when required. In contrast, BAT uses its stored fat to generate heat by oxidation of FFAs to maintain the body temperature [51]. In published studies, Plin5 is highly expressed in BAT but barely detectable in WAT [13]. The changes in LDs-protein gene expression included Plin1-5, suggesting that LDs experience a different adaptation to cold exposure in WAT and BAT cells. Prolonged cold exposure, which also induces the appearance of brown-like adipocytes in mice WAT depot, was accompanied with the enhancement in Plin5 expression [52]. However, arguing against an important role of Plin5 in BAT thermogenesis is the finding that Plin5^−/−^ and WT littermates have similar tolerance to the cold [22]. In the research of Tansey et al.., it consumed equal amounts of food, but the adipose tissue mass in the null animals was reduced to approximately 30% of that in WT. Meanwhile, the Plin5^−/−^ adipocyte showed higher basal lipolysis rate, thus proving that the perilipin knockout reduced the protective effect on LDs [53]. The biochemical pathways involved in obesity resistance in Plin5^−/−^ mice may provide a potential direction for obesity treatment.

## 3. The Concrete Role of Plin5 in Mitochondria

### 3.1. Pathophysiological Role of Plin5 in Mitochondria

Lipid droplet and mitochondria are important organelles involved in lipid metabolism and energy homeostasis. Cumulative research has shown that physical contact between the two organelles is important for their function, and Plin5 has been found to mediate this contact [54]. Phosphorylated Plin5 can migrate to nucleus to promote PGC-1*α* co-activator function by disinhibiting SIRT1 deacetylase activity, enhancing transcription of mitochondrial function, and reinforcing fatty acid metabolism [55]. Furthermore, it is suggested that Plin5 can recruit mitochondria to lipid droplets through a C-terminal region in a variety of cell and tissue types, including Chinese hamster ovary cells, AML12, HL-1 cells, primary brown fat cells, INS1 cells, and mouse heart cells [56], and Plin5 may mediate the interaction between lipid droplets and mitochondrial oxidative tissue [42]. The contact site between mitochondria and lipid droplet usually called peri-droplet mitochondria (PDM) is an important location for mitochondria to regulate lipid droplet storage and oxidation, and it can promote expansion of lipid droplets [57]. Plin5 increased the number of PDM by recruiting mitochondria to lipid droplets, which indirectly proved that Plin5 could promote the expansion of lipid droplets through PDM. To sum up, Plin5 can enhance mitochondrial function by increasing the role of PGC-1*α*. Moreover, Plin5 can promote the structural connection between lipid droplets and mitochondria, promoting the expansion of lipid droplets.

### 3.2. The Relationship of Plin5 and Mitophagy

Autophagy is a process for degradation of long-lived or injured organelles and proteins which involves vacuolar isolation of intracellular components and their targeted lysosomal degradation, promoting cellular responses to stress conditions including starvation and pathological stresses such as oxidative stress [58, 59]. There is growing evidence that autophagy, in addition to being a massive nonselective degradation mechanism, can selectively remove damaged mitochondria in order to promote mitochondrial turnover, a process known as “mitophagy” [60, 61]. The term “mitophagy” was first coined in 2005, and it can be divided into three types: type 1 usually involves phosphatidylinositide 3-kinases (PI3K) and is closely related to mitochondrial division. Under starvation conditions, preautophagic vesicles form cup-shaped phagocytic vesicles and then wrap mitochondria, resulting in depolarization of mitochondrial outer membrane and hydrolysis of autophagic vesicles in lysosomes. Type 2, in which damaged mitochondria bind to autophagosomes containing microtubule-associated protein 1 light chain 3(LC3), depolarization occurs without the involvement of PI3K and has nothing to do with mitochondrial division. Type 3 is also called micromitochondrial autophagy; oxidized mitochondrial protein forms mitochondria-derived vesicles (MDVs) through budding, and the vesicles are gradually fused into poly-vesicles, which are hydrolyzed by lysosomes into mitochondrial fragments [62, 63]. Mitophagy is involved in the occurrence and development of many diseases. Serine/threonine kinases, PTEN-induced putative kinase 1(PINK1), and E3 ubiquitin ligases, Parkin, are the most classical mechanisms in the study of mitophagy; PINK1 and Parkin mutations can lead to the accumulation of damaged mitochondria, which further promotes the occurrence of neuronal degeneration and ultimately leads to Parkinson's disease [64]. Expression of Parkin is also lost in many types of cancer, and overexpression of Parkin in breast and glioma cells inhibits cellular proliferation. Similar to Parkin, overexpression of PINK1 is thought to attenuate the growth of glioblastoma [65]. Saito et al. showed that Rab9 gene-mediated mitophagy maintained mitochondrial homeostasis through the Ulk1/Rab9/Rip1/Drp1 pathway in ischemic environment. However, knockout of Rab9 gene can inhibit mitophagy and aggravate myocardial injury caused by ischemia [66]. Mitophagy is involved in the occurrence of many diseases, but its mechanism is still unclear and needs further exploration. In a murine model under both fasting and refeeding conditions, Plin5 was required for the induction of autophagy during fasting, which contributed to its anti-inflammatory effects. The ability of Plin5 to promote autophagy and prevent inflammation was dependent upon signaling through SIRT1, which is known to be activated in response to nuclear Plin5 under fasting conditions [67]. Plin5 is a chaperone-mediated autophagy (CMA) substrate; its degradation through CMA is required for LD breakdown. The reduced activity of CMA failed to degrade Plin5 and other perilipin proteins (which are substrates of CMA), inhibited LD breakdown, and caused steatosis in hepatocytes [68]. What is more, mitochondrial autophagy can balance lipid generation by regulating lipid biosynthesis and decomposition to prevent the development of fatty liver, so effective activation of mitochondrial autophagy can be developed to treat fatty liver disease [69].

## 4. Upstream Factors Regulating Plin5

Previous studies have shown that many factors can influence the expression of Plin5 in the heart, liver, and skeletal muscle (Table 1). First, many molecules play crucial roles in Plin5 expression, such as peroxisome proliferator-activated receptor (PPAR)-*α*/*δ* [14], C/EBP-*α* [14], Curcumin, PGC-1*α* [42], lipocalin-2 (LCN2) [70], SREBP2 [71], miR-370, and ROS. PPAR-*α* agonist is able to increase the expression of Plin5 [16]. WY-14643, a PPAR-*α* agonist, can restore Plin5 expression in hypoxic cardiomyocytes [27]. C/EBP-*α* promotes the transcription of Plin5 gene in porcine [72]. Recent findings show that palmitate and PPAR agonists induced Plin5 expression in INS-1 cells in vitro [73]. Curcumin increases Plin5 gene expression to recover LD formation and lipid accumulation in activated hepatic stellate cells [74]. Basal Plin5 expression was significantly reduced in LCN2^−/−^ hepatocytes. Plin4 ablation also reduces Plin5 expression in mice, leading to decreased cardiac TG accumulation [75]. Low-density lipoprotein (LDL) strongly increases Plin5 expression in cardiomyocytes. Nonesterified fatty acids (NEFAs) are strong inducers of Plin5 transcription through PPAR activation [44]. Estrogen receptor-associated receptor (ERR)-*α* increases Plin5 expression by interacting with PPAR. Sulforaphane (SFN) decreases LD-associated protein Plin 2 and Plin5 expression that may be achieved by downregulating PPAR*γ* [76]. Oleic acid (OA) can induce Plin5 expression in HepG2 cells and in a dose- and time-dependent manner [77]. Overexpression of leptin in transgenic mice decreased Plin5 expression [78]. TNF-*α* decreased Plin5 expression and promoted lipolysis in the basal state. However, SREBP2 can inhibit the expression of Plin5 [71]. MiR-370 can downregulate Plin5, which in turn resulted in an increase in PPAR*α* and Bcl-2 expression to promote cardiomyocyte proliferation and inhibit cardiomyocyte cycle arrest and apoptosis [79]. ROS via JNK-p38-ATF signaling upregulated Plin5 expression and increased Plin5 to enhance lipid synthesis and to promote LD contact with mitochondria, which help cells to modulate stress response [80]. Second, many drugs can affect the expression of Plin5. Statins decrease the levels of Plin5 in the livers and primary hepatocytes, paralleled by a significant reduction in TG content. The transcription of Plin5 could be directly inhibited by SREBP2, which was upregulated by the cholesterol depletion of statins. One of the statins, atorvastatin, can promote PKA-mediated phosphorylation of Plin5 to reduce lipid accumulation in the liver [71]. PKA stimulation can enhance Plin5 phosphorylation to increase TG hydrolysis and direct FFAs to mitochondrial oxidation [81]. A functionally conserved PPRE site maps to the first intron of Plin5, and Plin5 expression can be induced in myocardium, skeletal, and liver by PPAR-*α* agonists, but also in WAT by pioglitazone, a PPAR-*γ* agonist. Some agonists, however, are not exclusive but can cross-activate different PPAR family members [16]. Third, some exogenous environmental stimuli can also affect its expression. Such as hypoxia, it can aggravate intracellular TG accumulation promoted by electro-negative LDL in cardiomyocytes via impairing Plin5 pathway [27]. Cold conditions also increase Plin5 expression [82]. And as LDs target protein, Plin5 expression is enhanced under physiological or pharmacological conditions that promote systemic FA elevation, e.g., fasting (liver and heart), endurance exercise (skeletal muscle), and chronic *β*3-adrenergic stimulation (liver). Exogenous FAs can also stimulate Plin5 expression in cell culture. One primary pathway involves the transcription factor family of PPARs [16]. Recently, some experiments on pigs suggested that variations in the Plin5 sequence might be linked to LIPE expression through a still poorly known regulative molecular process [83]. In an article by Yamada and Honma et al., they identified that Plin5 was upregulated in mice force-fed with fructose compared with those force-fed with glucose [84].

## 5. Downstream Factors Regulated by Plin5

The change of Plin5 expression can affect many physiological processes and molecular expression. Early studies have shown that Plin5 can prevent uncontrolled TG mobilization and excessive release of FFA [20] (Table 2) and act as a barrier to lipolysis. However, recent studies have found that PLIN proteins do not serve as lipolytic barriers but rather are docking sites for proteins facilitating selective lipase access under a variety of lipolytic conditions [85]. First, Plin5 can affect the genes correlated with lipid metabolism, such as SIRT1, CGI-58, HSL, and ATGL/ABHD5. Monounsaturated fatty acids (MUFAs) can bind to plin5 and activate its downstream target gene SIRT1, thus becoming the first known endogenous allosteric regulator of SIRT1 [86]. It is reported that Plin2, 3, and 5 all interact with HSL and ATGL [87]. Plin5 blocks ATGL-mediated lipolysis by competitively binding to CGI-58 and disrupting the interaction between CGI-58 and ATGL in the liver, thus inhibiting lipolysis and improving hepatic lipotoxicity [87]. Plin5 also expresses in myocardium and has been shown to interact with ATGL and its coactivator CGI-58 [20]. ATGL and its protein activator, *α*-*β*-hydrolase domain-containing 5 (ABHD5), each can bind to Plin5. ABHD5 potently activates ATGL, but this lipase-promoting activity is suppressed when ABHD5 is bound to Plin proteins on LDs [88]. The association of Plin5-ABDH5 complexes on lipid droplet surfaces was more stable than Plin5-ATGL complexes [89]. Second, Plin5 can influence the expression of antioxidant genes, such as PPAR-*α*, PGC1-*α*, Nrf2, and NAPDH. Plin5 deficiency can reduce myocardial lipid accumulation and upregulate PPAR-*α* and PGC1-*α* levels. The genes they control are involved in FA catabolism and mitochondrial oxidation [50]. Therefore, Plin5 deficiency increases the mobilization of stored lipids [34]. Moreover, Plin5 regulates the formation and stabilization of cardiac LDs, and it promotes cardiac steatosis without major heart function impairment, which may have been prevented by a strongly increased expression of oxidative-induced genes via Nrf2 antioxidative pathway [20]. Specially, membrane translocation of protein kinase C (PKC) and the assembly of NADPH oxidase 2 complex on the membrane were also suppressed. Diabetic Plin5-ablation mice are resistant to type 1 diabetes-induced heart malfunction due to the suppression of diacylglycerol/ceramide-PKC pathway and excessive ROS generated by NADPH oxidase [28]. Third, Plin5 plays a vital role in some signal paths, such as cAMP/GPR40, NF-*κ*b, phosphatidylinositol 3-kinase/protein kinase B (PI3K/AKT), phospho-Akt/phospho-glycogen synthase kinase-3*β*/nuclear factor erythroid 2-related factor 2Akt/GSK-3*β*/Nrf2, and MAPK. Upregulation of Plin5 in islets enhanced the augmentation of glucose-stimulated insulin secretion by FA and 8-Br-cAMP in G-protein-coupled receptor 40 (GPR40) and cAMP-activated protein kinase-dependent manners, respectively, implicating its role in the postprandial insulin secretion [90]. Interestingly, Plin5 deficiency promotes atherosclerosis progression through accelerating inflammation, apoptosis, and oxidative stress, which was linked with the activation of PI3K/AKT and mitogen-activated protein kinases (MAPKs) pathways [34]. In accordance with this, Plin5 plays an important role in protecting against HG-induced apoptosis, oxidative stress, and inflammation in podocytes via modulation of Akt/GSK-3*β*/Nrf2 signaling [91]. Moreover, Plin5 alleviates myocardial ischemia/reperfusion (MI/R) injury by reducing oxidative stress through increasing phosphorylation of PI3K/Akt to inhibit the lipolysis of LDs [26]. What is more, Plin5 can affect the fibroblast growth factor 21 (FGF21) expression. Plin5-driven LD accumulation in skeletal muscle stimulates the expression of FGF21. Upregulation of Plin5 drives the FGF21 gene expression in fast-twitch fibers and exhibits metabolically protective roles in skeletal muscle [91]. However, the detailed roles and mechanisms of Plin5 to regulate lipid turn over are far from clear yet. In particularly, we should pay more attention to the different modifications of Plin5 and the following changes in lipid homeostasis.

## 6. Prospect

Abnormal lipid metabolism plays an important role in the pathophysiology of diabetes, cardiovascular disease, liver disease, and its complications. Lipid metabolism and glucose metabolism disorders affect each other and together become the culprit of diabetic cardiovascular complications. Active lipid-lowering treatment in diabetic patients can significantly reduce the incidence of cardiac dysfunction and mortality. We proposed a hypothesis to connect all the pathways and to simulate the probable process of Plin5-related cardio-protection in diabetes. As one of the lipid-associated protein family members, Plin5 plays an important regulatory role in lipid deposition and orderly arrangement. In the basal state, perilipin anchored to the lipid droplet surface as a physiological barrier prevented soluble lipase from reaching the lipid droplet, rendering the TG unhydrolyzed by lipase, whereas phospholipidated by PKA upon lipolysis stimulation, leaving soluble lipase phosphoric acid translocated to the surface of LDs, at which point it colocalized with the phosphorylated perilipin on the surface of the LDs to stimulate lipolysis. It has been shown that ATGL plays a major role in basal lipolysis. Therefore, ATGL is speculated to be related to lipofuscin. There may be some unknown interaction between the proteins which could be another starting point to study perilipin and lipid metabolism. Plin5 can be seen as a connecting bridge between the mitochondrion and FFAs metabolism. Further studies are needed to clarify the role of Plin5 in recruiting and attaching mitochondria to the surface of LD and to determine which signaling pathways are involved in the regulation of mitochondrial localization of Plin5. In particular, the relationship between Plin5 and mitophagy may provide new ideas for the treatment of many diseases. As residual products of excessive FFA *β*-oxidation, ROS contribute to the development of diabetic cardiomyopathy and diabetic vascular complications. Recent findings have supported that Plin5 reduces ROS by sequestering FFAs from excessive oxidation and even is a critical regulator of lipid uptake and lipolysis. Therefore, we suppose that Plin5 may therefore represent a novel therapeutic drug target for the treatment of those diseases related to elevated fat accumulation and steatosis, for further understanding of abnormal body fat distribution, insulin resistance, consequently, opening up a new direction, providing new ideas to explore the development of this kind of drugs. We think that most of the studies on Plin5 were undertaken in cell systems and transgenic mice, but some cell systems lack other lipid metabolism proteins which may be necessary to faithfully replicate the protein-protein interactions required for in vivo lipolysis and other metabolic functions. The role of Plin5 in metabolic disease remains perplexing, owing to the lack of concordance between studies using similar experimental designs and interspecies differences. We are only beginning to delineate Plin5 responses to environmental/physiological situations, and further studies will provide a clearer picture of Plin5's functions in physiological and pathophysiological states in vivo.

**Figure 2:**
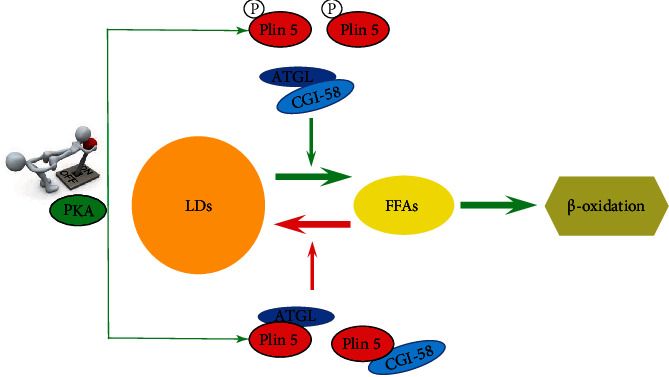
Plin5 serves as a bidirectional switch for FFAs metabolism.

Overall, Plin5 serves as a bidirectional switch in oxidative tissues, and this knowledge could lead to new avenues of therapy and prevention of diabetic cardiomyopathy. At present, the data indicates that a complex network of signaling mechanisms is involved in Plin5 mediation (Figure 2). Its numerous regulators and signaling pathways provide researchers with many chances to explore its mechanism. However, there are many unsolved issues regarding the function of Plin5 in the heart. Undoubtedly, more work is needed to understand the role of Plin5 in cardiomyocyte biology before it can be considered as a valuable therapeutic target for translational studies of diabetic cardiomyopathy.

## Figures and Tables

**Table 1:** Upstream factors regulating Plin5.

Upstream mediators	Effect target	Upstream regulation effect	First author, year, and reference no.
Molecules			
PPAR-*α*/*δ*	Muscle	Activation of PPAR-*α/δ* can increase the expression of Plin5	Bindesbøll C et al., 2012 [44]
C/EBP*α*	Adipocyte tissue, liver	C/EBP*α* promotes transcription of the porcine Plin5 gene	Zhou L et al., 2013 [72]
SREBP2	Liver	SREBP2 can inhibition the expression of Plin5	Asimakopoulou A et al., 2014 [70]
LCN2	Liver	Basal expression of Plin5 was significantly reduced in Lcn2^−/−^ cells	Gao X et al., 2017 [93]
Plin4	Heart	Plin4 ablation can reduce Plin5 expression at both mRNA and protein levels	Chen W et al., 2013 [75]
PKA	Heart, liver	Protein kinase A (PKA)-stimulation can enhance Plin5 phosphorylation	Pollak NM et al., 2015 [81]
LDL	Cardiomyocytes	LDL (-) strongly induces Plin5 mRNA expression and protein levels	Bindesbøll C et al., 2012 [44]
Environmental			
Hypoxia	Cardiomyocytes	Hypoxia can impair Plin5 upregulation	Revuelta-López E et al., 2015 [27]
Fasting	Liver, heart	Plin5 expression is enhanced	Kimmel AR et al., 2014 [16]
Chronic *β*3-adrenergic stimulation	Liver	Plin5 expression is enhanced	Kimmel AR et al., 2014 [16]
Endurance exercise	Skeletal muscle	Plin5 expression is enhanced	Kimmel AR et al., 2014 [16]

**Table 2:** Downstream factors regulated by Plin5.

Downstream mediators	Effect target	Downstream regulation effect	First author, year, and reference no.
CGI-58/ATGL	Cardiomyocyte, liver, adipocyte tissue, muscle	Plin5 competitively binds to CGI-58 and disrupting the interaction between CGI-58 and ATGL	Wang C et al.. 2015 [35] Pollak NM et al., 2013 [20] Sanders MA et al., 2015 [88] Mason RR et al., 2014 [39]
HSL	Adipocyte tissue	Plin5 interacts with HSL	Macpherson RE et al., 2013 [87]
PPAR*α*/PGC1-*α*	Muscle	Overexpression of Plin5 promotes expression of genes under control of PPAR*α* and PGC-1*α*	Bosma M et al., 2013 [50]
	Liver, heart	Plin5 decreases expression of PPAR*α* target genes	Trevino MB et al., 2015 [94] Wang H, et al.,2013 [21]
NF-E2-related factor 2	Heart	Plin5 increases expression of oxidative-induced genes via NF-E2-related factor 2 antioxidative pathway	Wang H et al., 2013 [21]
FGF21	Muscle	Upregulating the Plin5 level drives expression of the FGF21 gene	Harris LA et al., 2015 [41]
cAMP/GPR40	Islet	Ad-Plin5 enhanced glucose-stimulated insulin secretion in GPR40- and cAMP-activated protein kinase- dependent manners	Trevino MB et al., 2015 [90]
PKC/NAPDH	Heart	Plin5-KO suppresses diacylglycerol/ceramide-PKC pathway and NADPH oxidase	Kuramoto K et al., 2014 [28]
NF-*κ*B	Artery	I*κ*B*α*/NF-*κ*B pathway was activated in Plin5^−/−^	Zhou PL et al., 2017 [34]
MAPK	Aortic tissue	Plin5-/- activates PI3K/AKT and MAPKs pathways	Zhou PL et al., 2017 [34]
PI3K/AKT	Cardiomyocytes	Plin5-null decreases phosphorylation of PI3K/AKT	Zheng P et al., 2017 [26]
